# High invasion potential of *Hydrilla verticillata* in the Americas predicted using ecological niche modeling combined with genetic data

**DOI:** 10.1002/ece3.3072

**Published:** 2017-05-30

**Authors:** Jinning Zhu, Xuan Xu, Qing Tao, Panpan Yi, Dan Yu, Xinwei Xu

**Affiliations:** ^1^ National Field Station of Freshwater Ecosystem of Liangzi LakeCollege of Life SciencesWuhan UniversityWuhanChina

**Keywords:** climate change, ecological niche models, genetic lineage, *Hydrilla verticillata*, invasive species, niche shift

## Abstract

Ecological niche modeling is an effective tool to characterize the spatial distribution of suitable areas for species, and it is especially useful for predicting the potential distribution of invasive species. The widespread submerged plant *Hydrilla verticillata* (hydrilla) has an obvious phylogeographical pattern: Four genetic lineages occupy distinct regions in native range, and only one lineage invades the Americas. Here, we aimed to evaluate climatic niche conservatism of hydrilla in North America at the intraspecific level and explore its invasion potential in the Americas by comparing climatic niches in a phylogenetic context. Niche shift was found in the invasion process of hydrilla in North America, which is probably mainly attributed to high levels of somatic mutation. Dramatic changes in range expansion in the Americas were predicted in the situation of all four genetic lineages invading the Americas or future climatic changes, especially in South America; this suggests that there is a high invasion potential of hydrilla in the Americas. Our findings provide useful information for the management of hydrilla in the Americas and give an example of exploring intraspecific climatic niche to better understand species invasion.

## 1 INTRODUCTION

Species’ distributions are affected by both ecological and historical factors (Brown, 1995; Gaston, 2003; MacArthur, 1972). Climatic conditions are assumed to be the critical environmental control for the distribution of species on the large spatial scale, and ecological conditions limit the distributional potential of species (Brown & Pavlovic, 1992; Peterson, 2003). Climate change has a profound influence on the range of expansion and contraction of species, and may result in extinction of species (Chen, Hill, Ohlemüller, Roy, & Thomas, 2011; Parmesan & Yohe, 2003; Thomas et al., 2004; Thuiller, Lavorel, Araújo, Sykes, & Prentice, 2005), which in turn may create potential consequences on biodiversity and ecosystem functioning (Balvanera et al., 2006). Ongoing processes of climate change show warming of a few degrees, but it involves reorganization of many aspects of climate, such as rainfall (Houghton et al., 2001; Karl, Knight, Easterling, & Quayle, 1996). The twentieth century experienced the strongest warming trend of the last millennium with average temperatures rising by about 0.6°C (Jones, Osborn, & Briffa, 2001). For the future, IPCC (2007) predicts an ongoing increase of about 2.5–6.5°C in the mean annual temperature during the next 100 years and a shift in rainfall events. Understanding the impact of climatic change on species’ distribution will facilitate the prediction of how species respond to future climate change.

Ecological niche models (ENMs) are effective tools to characterize the spatial distribution of suitable areas for species. Applications of ENMs include characterizing full geographical distribution of species (Guisan et al., 2006), anticipating the existence of undescribed species (Raxworthy et al., 2003), estimating the invasive potential of species (Jiménez‐Valverde et al., 2011; Peterson, 2003; Thuiller, Richardson, et al., 2005), and forecasting the effect of climate change on species’ distributions (Araújo, Pearson, Thuiller, & Erhard, 2005; Peterson, 2011). Predicting the potential distribution of invasive species is especially important because invasive species have the potential to spread and affect native ecosystems (Lockwood, Cassey, & Blackburn, 2005); also, biological invasions are recognized as one of the most serious threats to global biodiversity and can cause economic and ecological problems (Sala et al., 2000). To predict the potential geographical extent of invasions, the classical approach is to use training models in the native range and project them to the invasive range based on the assumption of niche conservatism (Pearman, Guisan, Broennimann, & Randin, 2008; Wiens et al., 2010). However, this approach could be hampered if niche shifts have occurred in some species between their native and invasive ranges (e.g., Broennimann et al., 2007; Fitzpatrick, Weltzin, Sanders, & Dunn, 2007). An alternative approach with improved prediction is to pool occurrence data from all ranges when fitting models (Broennimann & Guisan, 2008).

Invasive species in aquatic habitats often have a greater impact on ecosystems than those in terrestrial ecosystems (Vilà et al., 2010) and invasive aquatic plants should be of great concern because they have the capacity to fundamentally alter ecosystem structure and functioning (Brundu, 2015; Chamier, Schachtschneider, Le Maitre, Ashton, & Van Wilgen, 2012; Kelly et al., 2014; Zedler & Kercher, 2004). Here, we focused on the invasion of a widespread submerged plant *Hydrilla verticillata* (L.f.) Royle (hydrilla). This species is native to Asia and Australia and invasive to North and South America (Cook, 1985; Cook & Lüönd, 1982; Sousa, Thomaz, Murphy, Silveira, & Mormul, 2009), but it remains contentious that Europe and Africa are native areas (Cook & Lüönd, 1982; Overholt et al., 2008). In North America, hydrilla covers much of the eastern, southern, and far western parts of USA and has become a noxious weed since its introduction into Florida in the 1950s (Cook, 1985; Cook & Lüönd, 1982). In South America, hydrilla was first found in southeast Brazil in 2005 and spread quickly in the Paraná River (Thomaz et al., 2009). Hydrilla infestations not only cause economic damages because dense surface mats decrease water quality, block irrigation systems, and hinder navigation, fisheries, and leisure activities (Monterroso, Binimelis, & Rodríguez‐Labajos, 2011), but they also become an ecological concern as hydrilla changes species composition and decreases biodiversity in native aquatic ecosystems (Mormul, Thomaz, Higuti, & Martens, 2010; Posey, Wigand, & Stevenson, 1993; Theel, Dibble, & Madsen, 2007). Two biotypes of hydrilla (i.e., dioecious and monoecious) have been recognized in the USA (Cook & Lüönd, 1982), and historical accounts suggest that they were separately introduced. The dioecious biotype was introduced into Florida in 1959 (Blackburn, Weldon, Yeo, & Taylor, 1969), and the introduction of the monoecious biotype occurred in Delaware in 1976 (Steward, Van, Carter, & Pieterse, 1984). Studies using RAPDs and sequences of chloroplast DNA suggested that the dioecious biotype was from the Indian subcontinent, and the monoecious biotype was from South Korea (Madeira, Coetzee, Center, White, & Tipping, 2007; Madeira, Van, Steward, & Schnell, 1997). Sequences of hydrilla samples from Brazil were homogeneous and grouped with the US dioecious hydrilla, suggesting that they originate from North America (Lucio LC, unpublished data, GenBank accession number: KP263390–KP263413). Recently, a phylogeographical study revealed that the southern part of East Asia and eastern China were also possible origination areas for the US dioecious and monoecious hydrilla, respectively, and both biotypes belonged to the same genetic lineage, which was one of the four genetic lineages in hydrilla (Zhu, Yu, & Xu, 2015). The invasion potential of hydrilla in North America has been predicted via ENMs in two previous studies (Barnes et al., 2014; Peterson, 2003). However, only occurrence data for the native range were used, and distribution ranges of different genetic lineages were not considered in these two studies. Based on three previous genetic studies with broad sampling (Benoit, 2011; Madeira et al., 2007; Zhu et al., 2015), four genetic lineages were identified in hydrilla, of which each has distinct distribution range, and only one lineage has been introduced into Americas until now. Many successful invasions were associated with the occurrence of multiple introductions, combining genotypes from differentiated source populations (Bock et al., 2015; Bossdorf et al., 2005; Dlugosch & Parker, 2008). Therefore, we can clarify the invasive potential of hydrilla in the Americas by comparing two invasive situations: One is only the introduced lineage invading the Americas and another is all four lineages invading the Americas. Furthermore, measured by species distribution modeling based on local and climatic variables, niches of lake macrophytes including submerged species were found not to be conserved over space and time (Alahuhta, Ecke, Johnson, Sass, & Heino, 2017). This means that hydrilla's distribution in the Americas may change in the future in response to global climatic change.

In this study, we predict the invasive potential of hydrilla in North America and South America combining an ENM with the results of previous genetic studies. Our aims are to (1) test whether the niche shifted or was conserved after invasion in North America, (2) compare climatic niches in a phylogenetic context and measure the invasive potential of hydrilla in Americas, and (3) predict the distribution of hydrilla in the Americas in the future in response to changes in temperature and rainfall associated with global warming.

## 2 MATERIALS AND METHODS

### 2.1 Data collection

We obtained occurrence data of hydrilla from the Chinese Virtual Herbarium (CVH, http://www.cvh.org.cn/), Australia's Virtual Herbarium (AVH, http://avh.chah.org.au/), the Global Biodiversity Information Facility (GBIF, http://www.gbif.org/), and previously published papers (listed in Table S1). Duplicate and anomalous records were removed to reduce potential errors. A total of 693 occurrence data were kept for subsequent analysis (Table S1). Based on previous genetic studies (Benoit, 2011; Madeira et al., 2007; Zhu et al., 2015), genetic information from 412 sites was identified (Table S1) and their occurrences were depicted in Figure S1.

We obtained 19 layers of bioclimatic variables from the WorldClim Global Climate Data (http://www.worldclim.org) at a resolution of 2.5 arc‐minutes. The 19 variables are derived from combinations of temperature, precipitation, and seasonality records and are often used to predict the geographical distributions of plants, including terrestrial (Loiselle et al., 2008) and aquatic species (Alahuhta et al., 2017). The future data (2070) are climate projections from global climate models (GCMs) for four representative concentration pathways (RCPs) that characterize radiative forcing values in 2100 relative to preindustrial concentrations and range from 2.6 to 8.5 Watts per square meter. Only the maximum RCP 8.5 was used in this study.

### 2.2 Niche conservatism

Among the 412 sites with genetic information, 358 belong to the introduced lineage, 281 in North America and 74 in the native range. We therefore can evaluate niche conservatism in the invasion of hydrilla by comparing niches based on the occurrence data of the same genetic lineage in the native range and North America.

We characterize the environmental niche by the first two axes of a principal component analysis (PCA) built with the bioclimatic variables, which are referred to as PCA‐env in Broennimann et al. (2012). The species occurrence was converted into smooth densities using a kernel function and plotted in the gridded environmental space. Observed niche overlap between native and invasive ranges was qualified using Schoener's index of niche breadth (*D*) (Broennimann et al., 2012; Schoener, 1970). This statistics (*D*) ranged from 0 to 1, with 0 indicating no niche overlap and 1 indicating total overlap. To statistically evaluate niche overlap, we subsequently conducted a niche similarity test. The niche similarity test was calculated by comparing the observed niche overlap against a null distribution of 100 simulated overlap values (Broennimann et al., 2012). When the value of observed overlap was greater than the simulated one, the null hypothesis was rejected (*p* < .05), which indicated that the niches of the species were more similar than would be expected at random.

We also calculated niche expansion (new environmental requirements observed in the exotic niche), niche stability (proportion of the native niche observed in the exotic niche), and niche unfilling (proportion of the native niche not occupied in the exotic niche) (Guisan, Petitpierre, Broennimann, Daehler, & Kueffer, 2014; Petitpierre et al., 2012) to measure species niches. These measurements were obtained by using 95% of the intersection area between the native and invaded environmental space to control the environmental outliers (Petitpierre et al., 2012).

All these analyses were performed in R 3.1.2 (R Core Team, 2014). Niche comparisons between the native and invasive ranges were conducted with the *ecospat* package (Broennimann et al., 2014).

### 2.3 Ecological niche modeling

We applied a maximum entropy algorithm to predict the potential current (average for 1950–2000) and future (2070) distributions of hydrilla using MaxEnt 3.3 (Phillips, Anderson, & Schapire, 2006; Phillips & Dudík, 2008). Twenty‐five percent of the occurrence data were used for testing and the remaining 75% were used for training the model. The analysis was run with the default parameters. The area under curve (AUC) of the receiver‐operating characteristic was used to measure the model's performance in predicting environmentally suitable areas (Fielding & Bell, 1997; Marzban, 2004; Peterson, Papeş, & Soberón, 2008). An AUC value greater than 0.9 indicates high reliability of the model, while a value greater than 0.5 indicates that the model discriminates better than random (Manel, Williams, & Ormerod, 2001; Phillips et al., 2006; Swets, 1988).

Niche shift was detected in the invaded area of hydrilla (see Section 3); therefore, we predicted the invasion potential of hydrilla in the Americas by pooling occurrence data from both native and invasive ranges, which was suggested by Broennimann and Guisan (2008). Two occurrence datasets were used here: One dataset is comprised of the occurrence sites of the introduced genetic lineage in the native and invasive ranges and another contains all sites of hydrilla in the world. All results obtained from the ENMs were visualized in ArcMap 10.0 (Environmental Systems Research Institute, ESRI).

## 3 RESULTS

### 3.1 Niche conservatism

**Figure 1:**
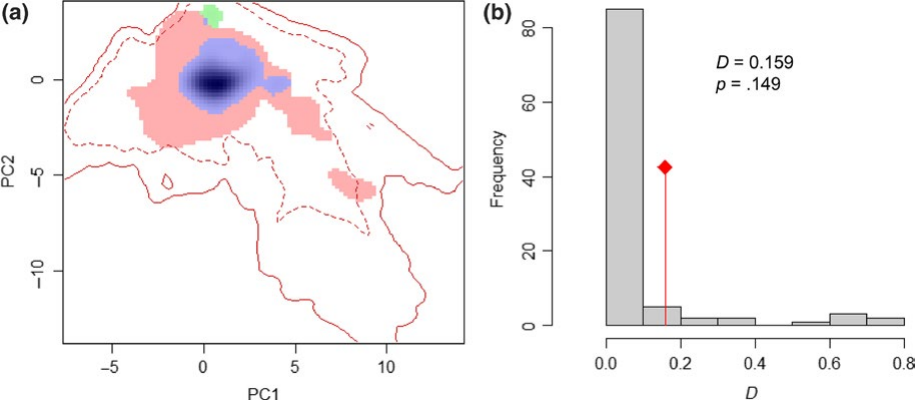
(a) Niche occupancy between native and invasive ranges based on the first two axes of principal component space derived from climatic data. Darker areas represent greater occupancy. The solid contour line is 100% suitable niche space, and the dashed line is 50% of the niche space. Green, red, and blue areas represent the niche of the native region, invasive region, and the shared niche, respectively. (b) Histogram of the niche similarity test between invasive and native ranges. The red lines with a diamond represent the observed niche overlap, and gray bars represent simulated niche overlaps. The *D* and *p* values of the test are shown

The first two PCA axes generated based on the climatic space explained 72.48% of the original environmental variation. The contribution of each original variable on each axis of the analysis is listed in Table 1, and the three most important variables were the min temperature of coldest month (Bio6), mean temperature of coldest quarter (Bio11), and annual mean temperature (Bio1). Visualization of the environmental spaces of hydrilla in the native and invasive ranges is represented in Figure 1a. When quantified, the values of niche overlap (*D*) are 0.159 (Figure 1b). The null hypothesis of niche similarity test was not rejected (*p *=* *.149), indicating that the climate niches of hydrilla in the two ranges were not more similar than would be expected at random. The median values of niche expansion (0.419) and niche stability (0.581) as well as the low value of niche unfilling (0.017) suggested that hydrilla in the invasive range not only occupied the major of native niche but also expanded considerably beyond their native niche (Figure 1a). These results showed the occurrence of a niche shift in hydrilla in its invasion process.

**Table 1:** Relative contributions of climatic variables to the first two axes in the PCAs

Variables	Description of the variable	PC1	PC2
Bio1	Annual mean temperature	0.281	0.208
Bio2	Mean diurnal range	−0.189	0.155
Bio3	Isothermality	0.230	−0.081
Bio4	Temperature seasonality	−0.267	−0.023
Bio5	Max temperature of warmest month	0.170	0.316
Bio6	Min temperature of coldest month	0.299	0.188
Bio7	Temperature annual range	−0.279	0.049
Bio8	Mean temperature of wettest quarter	0.219	0.233
Bio9	Mean temperature of driest quarter	0.264	0.156
Bio10	Mean temperature of warmest quarter	0.220	0.268
Bio11	Mean temperature of coldest quarter	0.293	0.157
Bio12	Annual precipitation	0.266	−0.223
Bio13	Precipitation of wettest month	0.242	−0.000
Bio14	Precipitation of driest month	0.158	−0.399
Bio15	Precipitation seasonality	−0.006	0.352
Bio16	Precipitation of wettest quarter	0.249	−0.023
Bio17	Precipitation of driest quarter	0.164	−0.398
Bio18	Precipitation of warmest quarter	0.194	−0.170
Bio19	Precipitation of coldest quarter	0.179	−0.337

### 3.2 Ecological niche modeling

**Figure 2:**
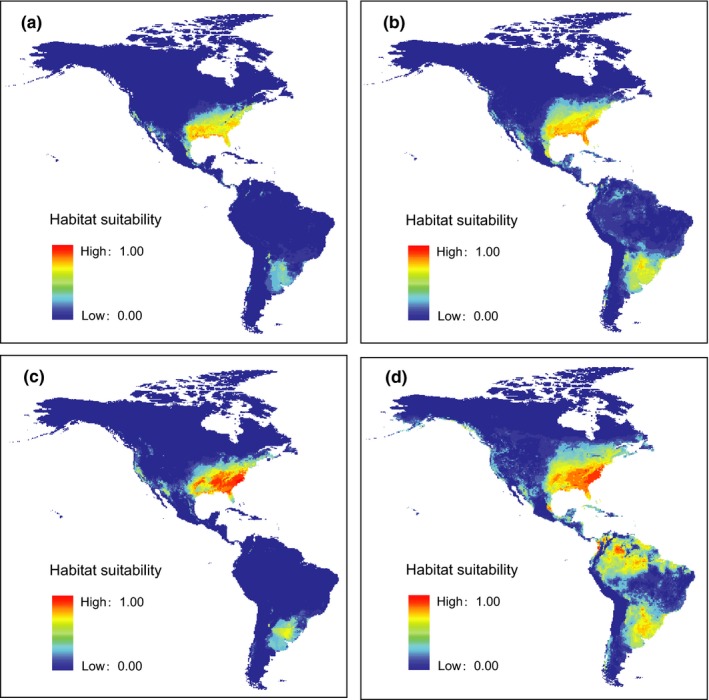
Ecological Niche Modeling for *Hydrilla verticillata* suitable habitat in the Americas under current and future climate conditions with different occurrence data. (a) Occurrence of the introduced genetic lineage from invasive and native ranges under current conditions; (b) occurrence of all four lineages in the world under current conditions; (c) occurrence of the introduced genetic lineage from invasive and native ranges under future conditions; (d) occurrence of all four lineages in the world under future conditions

For the introduced genetic lineage and all four lineages of hydrilla, the AUC values for the ENMs were 0.971 and 0.932, respectively, indicating that the model prediction was highly reliable. The probability maps of habitat suitability in the Americas under different conditions are presented in Figure 2. When considering probabilities greater than 0.5, the potential range of the introduced lineage in the Americas was 2,245,032 km^2^, while that of all four lineages was 3,961,073 km^2^ under the current conditions, with an increase of about 76% (Figure 2a,b and Table 2). In North America, only about 20% of the areas were increased, whereas in South America, the predicted distribution area of all four lineages is more than 50 times larger than that of the introduced lineage (Figure 2a,b and Table 2).

**Table 2:** Predicted distribution areas in North America and South America using ecological niche modeling based on different datasets under current and future conditions

Occurrence data	AUC	Current (km^2^)			Future (km^2^)		
		North America	South America	Americas	North America	South America	Americas
The introduced genetic lineage	0.971	2,220,946	24,086	2,245,032	2,663,668	330,592	2,994,260
All four genetic lineages	0.932	2,670,940	1,290,133	3,961,073	3,940,046	4,259,964	8,200,010

With increasing mean annual temperature and shifting in rainfall events under predicted climatic change in the future (2070), the potential distribution of hydrilla expanded in the Americas. Under the scenario modeled with RCP8.5, the predicted distribution area of the introduced lineage in 2070 spanned approximately 2,994,260 km^2^, which is about 1.3 times larger than the area spanned under the current condition (Figure 2a,c and Table 2). Among them, small changes occurred in North America, whereas the range expanded more than 13‐fold in South America (Figure 2a,c and Table 2). If all four genetic lineages invade the Americas, the climatic changes will promote hydrilla to expand on a larger scale. The predicted distribution area in 2070 was 8,200,010 km^2^, which is twice as large as those under the current condition (Figure 2b,d and Table 2). Among them, about 1.5‐ and 3.3‐fold range expansions were predicted in North America and South America, respectively (Table 2).

## 4 DISCUSSION

### 4.1 Niche shift in hydrilla

The latitudinal distribution of invasive populations in North America is broader than that of native populations that belonged to the same genetic lineage in Asia (Madeira, Jacono, & Van, 2000; Zhu et al., 2015; Figure S1). Furthermore, our analysis showed that niche shift occurred in hydrilla after its invasion into North America. As for the niche shift during the biological invasion, at least three explanations have been proposed. The first explanation is that the niche shift may be due to the absence of competitors and the ability to obtain more availability niches in new environment (Callaway & Maron, 2006; Mitchell et al., 2006). In native range, populations of the other three genetic lineages may act as competitors for the introduced genetic lineage, while they are absent in North America. It seems likely that the broader distribution of hydrilla in North America can be explained by the absence of competitors. However, some species in Hydrocharitaceae with similar ecological strategies and growth form as hydrilla occur in the Americas (e.g., *Elodea canadensis*,* Egeria densa*) and act as competitors (Hofstra, Clayton, Green, & Auger, 1999; Mony, Koschnick, Haller, & Muller, 2007). Therefore, the absence of competitors is probably not the likely explanation. A second possibility is that an evolutionary process or a different biotic environment is possible in nonnative ranges (Blossey & Notzold, 1995; Dietz & Edwards, 2006; Keane & Crawley, 2002). Hydrilla first invaded North America in the 1950s (Cook & Lüönd, 1982). Although rapid evolutionary changes during invasions occurred in many species and some cases happened in very short timescales (Cox, 2004; Lee, 2002; Whitney & Gabler, 2008), none occurred for submerged plants. Furthermore, the hypothesis of a conservative macroevolutionary pattern in submerged plants (Les, 1988; Barrett, Eckert, & Husband, 1993) makes this explanation not likely valid. The third explanation is that the niche shift is caused by the increasing genetic diversity from the potential invasion of other genetic lineages or genetic variation from genetic drift, variable, or hybridization (Cox, 2004; Dlugosch & Parker, 2008). The invasion of other genetic lineages of hydrilla into North America is denied according to the results of previous studies (Benoit, 2011; Madeira et al., 2007), whereas increasing genetic diversity through mutation is possible for hydrilla. In North America, only female plants were found in the dioecious biotype, which results in a lack of sexual reproduction, and only a few instances of sexually reproduction have been recorded for the monoecious biotype in the wild (Steward, 1993). Although successful artificial crosses between these two biotypes have been conducted, only a few cooccurrence sites were observed, and no seed production was reported in the field (Harlan, Davis, & Pesacrea, 1985; Steward, 1993). Therefore, the proportion of sexual reproduction should be very low for hydrilla in North America. Recently high genetic variation in neutral microsatellite loci was reported in dioecious hydrilla, which is due to somatic mutation (Grajczyk, 2009). Rapid clonal growth and high‐yield vegetative propagules in hydrilla (Langeland, 1996) provide ample opportunities for somatic mutations and potential dispersal from their point of origin (Grajczyk, 2009). It is a viable approach for hydrilla to increase its own genetic diversity through somatic mutations.

### 4.2 Invasion potential of hydrilla in Americas

Although hydrilla's sexual reproduction is very limited in North America, it can produce a variety of vegetative propagules to ensure its growth and establishment, including fragmentations, turions, and tubers, which make it spread rapidly and has become a serious weed (Langeland, 1996; Schmitz, Nelson, Nall, & Schardt, 1991). Balciunas and Chen (1993) suggested that hydrilla would expand its invasive range northward based on its current distribution in native and invasive ranges without considering its genetic background. Now we predict the invasion potential by comparing the possible ranges of the introduced genetic lineage and all four lineages using ENMs. The potential distribution of hydrilla in North America will expand a bit northward with an increase of about 20% but not too far toward northern latitudes (Figure 2a,b and Table 2). Dramatic changes occur in South America. Hydrilla recently invaded the Paraná River (the first record in June 2005) and spread rapidly along the margins of Paraná River (Sousa, 2011; Sousa et al., 2009). Hydrilla has high competitive potential in South America because it can outcompete native *Egeria densa*, which has very similar morphological and ecological features and often cooccurs in the same habitats as hydrilla, in most situations (Mony et al., 2007). If all four genetic lineages invade into South America, hydrilla will increase its potential range by more than 50 times, covering almost half of the entire Paraná River basin (Figure 2a,b and Table 2). Therefore, higher invasion potential of hydrilla is presented in South America.

Climate change will result in a range shift of species and drive populations to more suitable environments (Gottfried et al., 2012; Opdam & Wascher, 2004; Thuiller, 2007). A 3°C change in mean annual temperature corresponds to a shift in isotherms of approximately 300–400 km in latitude (temperate zone) or 500 m in elevation (Hughes, 2000). Our analyses revealed that the min temperature of coldest month, mean temperature of coldest quarter, and annual mean temperature were the three most important variables in predicting potential distribution of hydrilla; this suggests that low temperature a limiting factor. Previous studies found increased growth by increasing water temperature up to 33°C in growth chamber experiments (McFarland & Barko, 1987; Van, Haller, & Garrard, 1978), and an optimal temperature (36.5°C) for photosynthesis and measurable CO_2_ fixation in temperature as high as 44°C under laboratory conditions (Chen, 1989; Van, Haller, & Bowes, 1976). These results indicate that hydrilla is thermophilic. We thus can deduce that hydrilla will expand its range under increasing mean annual temperatures that may be caused by climatic change in the future. Our results showed that about a 20% increase in potential range occurred in North America, and more than a 13‐fold range expansion occurred in South America in 2070 under climatic change (Figure 2a,c and Table 2). In the situation where invasion occurred by all four lineages, the corresponding values were 1.5‐fold and 3.3‐fold range expansion in North and South America, respectively (Figure 2b,d and Table 2). Noticeably, in South America, the potential range was not limited in the southeastern region anymore, and it expanded into the northern region, including the Amazon River basin (Figure 2d). Therefore, in consideration of the possible invasion of hydrilla's new genetic lineages and climatic changes in the future, the invasion potential of hydrilla is high in the Americas, especially in South America. To avoid a new invasion, importing hydrilla plants from native countries into the Americas should be strictly forbidden.

## 5 CONCLUSION

Combined with the results of previous genetic studies (Benoit, 2011; Madeira et al., 2007; Zhu et al., 2015), our study reveals that niche shifts occur in the invasion of hydrilla in North America, and our findings point to a high invasion potential of hydrilla in both the Americas, especially in South America, in the situation where all four native genetic lineages invade the Americas or under conditions of future climatic changes. This study provides useful information for the management of hydrilla invasion and gives an example of exploring intraspecific climatic niche to better understand species invasion.

## ACKNOWLEDGEMENTS

This study was supported by grants from the National Natural Science Foundation of China to Xinwei Xu (31270265) and the Major Science and Technology Program for Water Pollution Control and Treatment to Dan Yu (2015ZX07503‐005). We thank the members of Dan Yu's group for their advice and field assistance.

## DATA ACCESSIBILITY

The BioClim variables of the studied samples were deposited in Dryad (http://dx.doi.org/10.5061/dryad.1v640).

## CONFLICT OF INTEREST

None declared.

## AUTHORS' CONTRIBUTIONS

XX and DY conceived the ideas; JZ, QT and PY collected the data; and JZ and XX analyzed the data and led to the writing.
