# Supplementary material for: High invasion potential of Hydrilla verticillata in the Americas predicted using ecological niche modeling combined with genetic data
Source: Ecol Evol. 2017 May 30;7(13):4982–90. doi: 10.1002/ece3.3072 (PMC5496529; doi:10.1002/ece3.3072)

Fig. S1 Distribution of four genetic lineages of Hydrilla verticillata in the world. Red dots are the introduced lineage, and blue, green, and yellow dots are the other three lineages. Grey dots show sites that lack genetic information.

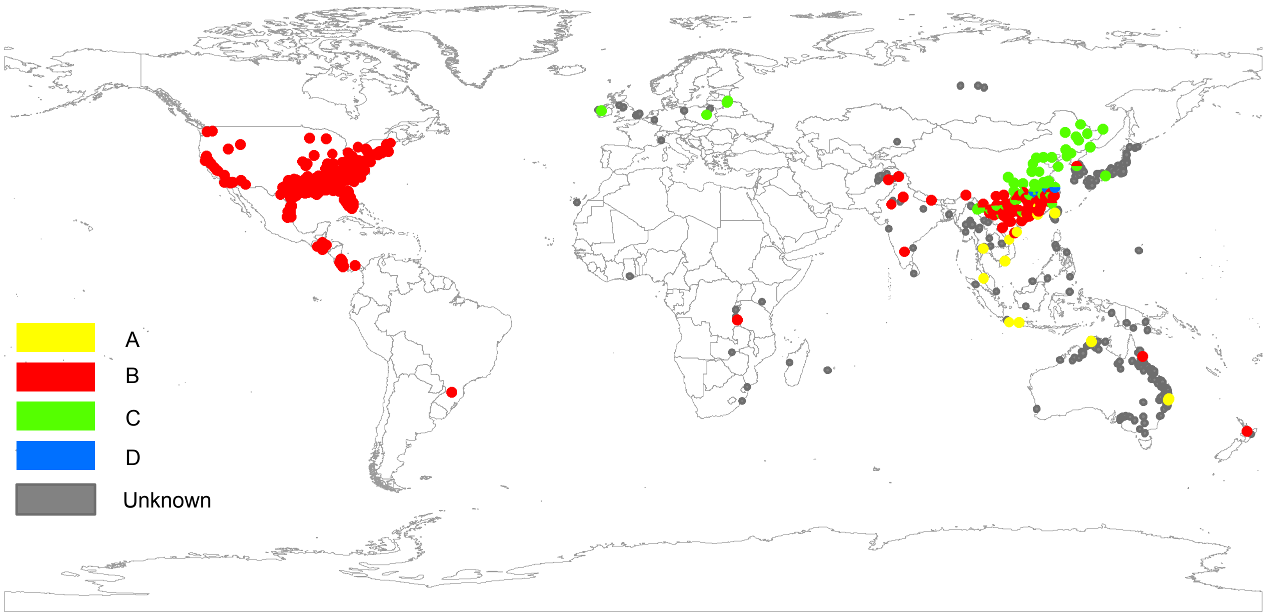

Supplement: Supplementary file 1 [file ECE3-7-4982-s001.pdf]
