# Supplementary material for: High invasion potential of Hydrilla verticillata in the Americas predicted using ecological niche modeling combined with genetic data
Source: Ecol Evol. 2017 May 30;7(13):4982–90. doi: 10.1002/ece3.3072 (PMC5496529; doi:10.1002/ece3.3072)
Supplement: Supplementary file 2 [file ECE3-7-4982-s002.docx]

**Table S1** Detailed occurrence records of *Hydrilla verticillata* used in this study. The genetic lineage was identified according to the listed references.

| Continent | Source | Genetic lineage | Latitude | Longitude |
| --- | --- | --- | --- | --- |
| Africa | Cook and Lüönd 1982 | Unknown | 6.08 | -1.01 |
| Africa | Cook and Lüönd 1982 | Unknown | -5.75 | 29.42 |
| Africa | Cook and Lüönd 1982 | Unknown | -29.72 | 31.09 |
| Africa | GBIF | Unknown | 27.10 | -16.10 |
| Africa | GBIF | Unknown | -15.75 | 28.17 |
| Africa | GBIF | Unknown | -3.58 | 29.35 |
| Africa | GBIF | Unknown | -25.73 | 32.67 |
| Africa | GBIF | Unknown | -1.25 | 36.83 |
| Africa | GBIF | Unknown | -18.70 | 44.71 |
| Africa | GBIF | Unknown | -20.93 | 55.61 |
| Africa | Madeira et al. 2007 | B | -6.50 | 29.83 |
| Asia | AVH | Unknown | -32.00 | 115.49 |
| Asia | Cook and Lüönd 1982 | Unknown | 19.55 | 73.09 |
| Asia | Cook and Lüönd 1982 | Unknown | 27.57 | 74.22 |
| Asia | Cook and Lüönd 1982 | Unknown | 44.53 | 75.49 |
| Asia | Cook and Lüönd 1982 | Unknown | 14.06 | 80.05 |
| Asia | Cook and Lüönd 1982 | Unknown | 6.76 | 80.24 |
| Asia | Cook and Lüönd 1982 | Unknown | 60.56 | 93.68 |
| Asia | Cook and Lüönd 1982 | Unknown | 60.56 | 98.69 |
| Asia | Cook and Lüönd 1982 | Unknown | 60.04 | 100.45 |
| Asia | Zhu et al. 2015 | B | 18.37 | 109.17 |
| Asia | Zhu et al. 2015 | A | 18.67 | 109.77 |
| Asia | Zhu 2015 | B | 21.48 | 101.57 |
| Asia | Zhu et al. 2015 | B | 21.60 | 108.00 |
| Asia | Zhu et al. 2015 | B | 21.72 | 107.39 |
| Asia | Zhu et al. 2015 | B | 21.84 | 111.24 |
| Asia | CVH | Unknown | 21.86 | 111.16 |
| Asia | CVH | Unknown | 21.96 | 100.45 |
| Asia | Zhu et al. 2015 | AB | 22.95 | 112.44 |
| Asia | Zhu et al. 2015 | B | 23.02 | 106.59 |
| Asia | Zhu 2015 | B | 23.13 | 113.34 |
| Asia | Zhu et al. 2015 | B | 23.27 | 106.00 |
| Asia | Zhu et al. 2015 | B | 23.46 | 103.31 |
| Asia | Zhu et al. 2015 | A | 23.63 | 115.70 |
| Asia | Zhu et al. 2015 | BC | 23.70 | 105.45 |
| Asia | Zhu et al. 2015 | B | 23.85 | 112.57 |
| Asia | Zhu et al. 2015 | B | 24.05 | 114.19 |
| Asia | Zhu et al. 2015 | B | 24.08 | 102.05 |
| Asia | Zhu 2015 | B | 24.20 | 108.14 |
| Asia | Zhu 2015 | B | 24.39 | 106.01 |
| Asia | Zhu et al. 2015 | B | 24.46 | 108.68 |
| Asia | Zhu et al. 2015 | B | 24.49 | 110.40 |
| Asia | Zhu et al. 2015 | BC | 24.53 | 111.32 |
| Asia | Zhu et al. 2015 | B | 24.62 | 115.84 |
| Asia | Zhu et al. 2015 | B | 24.63 | 102.88 |
| Asia | Zhu et al. 2015 | B | 24.65 | 116.19 |
| Asia | Zhu et al. 2015 | B | 24.76 | 113.31 |
| Asia | Zhu 2015 | B | 24.85 | 110.45 |
| Asia | Zhu et al. 2015 | B | 24.92 | 115.69 |
| Asia | Zhu et al. 2015 | B | 25.02 | 109.98 |
| Asia | Zhu et al. 2015 | BC | 25.12 | 102.09 |
| Asia | Zhu 2015 | C | 25.13 | 98.56 |
| Asia | Zhu et al. 2015 | B | 25.29 | 107.41 |
| Asia | Zhu 2015 | C | 25.36 | 98.63 |
| Asia | Zhu 2015 | B | 25.40 | 112.95 |
| Asia | Zhu et al. 2015 | B | 25.57 | 116.54 |
| Asia | Zhu et al. 2015 | BC | 25.67 | 100.21 |
| Asia | Zhu et al. 2015 | AB | 25.74 | 115.90 |
| Asia | Zhu 2015 | BC | 26.11 | 99.95 |
| Asia | Zhu et al. 2015 | BC | 26.20 | 105.87 |
| Asia | Zhu et al. 2015 | BC | 26.25 | 104.06 |
| Asia | Zhu et al. 2015 | B | 26.39 | 116.68 |
| Asia | Zhu et al. 2015 | BC | 26.43 | 106.30 |
| Asia | Zhu et al. 2015 | B | 26.51 | 107.17 |
| Asia | Zhu et al. 2015 | B | 26.56 | 115.21 |
| Asia | Zhu et al. 2015 | BC | 26.60 | 100.19 |
| Asia | Zhu 2015 | B | 26.63 | 107.56 |
| Asia | Zhu et al. 2015 | B | 26.83 | 116.86 |
| Asia | Zhu et al. 2015 | BC | 26.86 | 119.87 |
| Asia | Zhu et al. 2015 | B | 27.05 | 108.75 |
| Asia | Zhu et al. 2015 | B | 27.12 | 117.31 |
| Asia | Zhu et al. 2015 | B | 27.17 | 112.89 |
| Asia | Zhu et al. 2015 | B | 27.30 | 115.15 |
| Asia | Zhu et al. 2015 | B | 27.31 | 119.01 |
| Asia | Zhu et al. 2015 | B | 27.63 | 117.98 |
| Asia | Zhu et al. 2015 | B | 27.77 | 117.03 |
| Asia | Zhu et al. 2015 | B | 27.77 | 109.20 |
| Asia | Zhu et al. 2015 | B | 27.77 | 116.08 |
| Asia | Zhu 2015 | C | 27.90 | 109.32 |
| Asia | Zhu et al. 2015 | BC | 27.93 | 109.70 |
| Asia | Zhu 2015 | B | 28.35 | 112.16 |
| Asia | Zhu et al. 2015 | B | 28.75 | 120.21 |
| Asia | Zhu et al. 2015 | B | 28.96 | 103.90 |
| Asia | Zhu 2015 | C | 29.01 | 114.41 |
| Asia | Zhu et al. 2015 | B | 29.10 | 117.99 |
| Asia | Zhu 2015 | B | 29.10 | 120.54 |
| Asia | Zhu et al. 2015 | B | 29.12 | 110.42 |
| Asia | Zhu et al. 2015 | B | 29.24 | 106.09 |
| Asia | Zhu et al. 2015 | B | 29.25 | 95.23 |
| Asia | Zhu 2015 | CD | 29.26 | 115.90 |
| Asia | Zhu et al. 2015 | C | 29.32 | 117.15 |
| Asia | Zhu et al. 2015 | BC | 29.34 | 113.40 |
| Asia | Zhu et al. 2015 | BC | 29.53 | 119.31 |
| Asia | Zhu et al. 2015 | BCD | 29.98 | 111.77 |
| Asia | Zhu et al. 2015 | BC | 29.99 | 119.69 |
| Asia | Zhu et al. 2015 | CD | 30.22 | 116.57 |
| Asia | Zhu et al. 2015 | CD | 30.26 | 114.56 |
| Asia | Zhu et al. 2015 | C | 30.29 | 108.95 |
| Asia | Zhu 2015 | CD | 31.23 | 118.57 |
| Asia | Zhu 2015 | CD | 31.43 | 120.75 |
| Asia | Zhu 2015 | C | 31.46 | 115.09 |
| Asia | Zhu 2015 | C | 32.38 | 114.89 |
| Asia | Zhu et al. 2015 | C | 32.53 | 111.50 |
| Asia | Zhu 2015 | C | 32.71 | 108.95 |
| Asia | Zhu et al. 2015 | C | 33.01 | 117.38 |
| Asia | Zhu et al. 2015 | C | 33.29 | 118.83 |
| Asia | Zhu et al. 2015 | C | 34.34 | 107.37 |
| Asia | Zhu 2015 | C | 35.90 | 116.23 |
| Asia | Zhu et al. 2015 | C | 36.04 | 114.12 |
| Asia | Zhu et al. 2015 | C | 37.39 | 121.62 |
| Asia | Zhu et al. 2015 | C | 38.17 | 114.37 |
| Asia | Zhu et al. 2015 | C | 39.84 | 115.37 |
| Asia | Zhu et al. 2015 | C | 39.99 | 119.73 |
| Asia | Zhu et al. 2015 | C | 40.02 | 117.44 |
| Asia | Zhu 2015 | C | 40.47 | 124.11 |
| Asia | Zhu et al. 2015 | C | 41.24 | 125.36 |
| Asia | Zhu 2015 | C | 42.21 | 123.84 |
| Asia | Zhu et al. 2015 | C | 42.93 | 130.81 |
| Asia | Zhu et al. 2015 | C | 44.91 | 127.47 |
| Asia | Zhu 2015 | C | 45.97 | 126.72 |
| Asia | Zhu 2015 | C | 46.81 | 129.99 |
| Asia | Zhu 2015 | C | 47.19 | 123.68 |
| Asia | Zhu et al. 2015 | C | 48.14 | 134.48 |
| Asia | Zhu 2015 | C | 49.47 | 128.09 |
| Asia | GBIF | Unknown | 33.45 | 69.98 |
| Asia | GBIF | Unknown | 34.55 | 70.23 |
| Asia | GBIF | Unknown | 34.43 | 70.45 |
| Asia | GBIF | Unknown | 34.25 | 70.82 |
| Asia | GBIF | Unknown | 34.75 | 70.83 |
| Asia | GBIF | Unknown | 34.62 | 71.98 |
| Asia | GBIF | Unknown | 35.33 | 72.58 |
| Asia | GBIF | Unknown | 34.08 | 74.82 |
| Asia | GBIF | Unknown | 27.36 | 76.41 |
| Asia | GBIF | Unknown | 25.30 | 82.98 |
| Asia | GBIF | Unknown | 23.72 | 90.37 |
| Asia | GBIF | Unknown | 18.71 | 95.10 |
| Asia | GBIF | Unknown | 20.80 | 95.25 |
| Asia | GBIF | Unknown | 22.01 | 96.47 |
| Asia | GBIF | Unknown | 21.00 | 96.67 |
| Asia | GBIF | Unknown | 26.11 | 96.72 |
| Asia | GBIF | Unknown | 20.46 | 96.84 |
| Asia | GBIF | Unknown | 20.79 | 97.04 |
| Asia | GBIF | Unknown | 3.59 | 97.98 |
| Asia | GBIF | Unknown | 19.48 | 98.91 |
| Asia | GBIF | Unknown | 19.95 | 99.18 |
| Asia | GBIF | Unknown | 14.03 | 99.52 |
| Asia | GBIF | Unknown | 5.84 | 100.47 |
| Asia | GBIF | Unknown | 13.75 | 100.50 |
| Asia | GBIF | Unknown | 14.50 | 100.50 |
| Asia | GBIF | Unknown | 16.83 | 101.97 |
| Asia | GBIF | Unknown | 14.67 | 102.33 |
| Asia | GBIF | Unknown | 1.64 | 103.92 |
| Asia | GBIF | Unknown | 15.40 | 105.49 |
| Asia | GBIF | Unknown | -6.55 | 106.81 |
| Asia | GBIF | Unknown | -2.53 | 112.32 |
| Asia | GBIF | Unknown | 4.57 | 114.28 |
| Asia | GBIF | Unknown | 5.36 | 118.65 |
| Asia | GBIF | Unknown | 23.22 | 120.16 |
| Asia | GBIF | Unknown | 22.66 | 120.44 |
| Asia | GBIF | Unknown | 23.48 | 120.46 |
| Asia | GBIF | Unknown | 22.18 | 120.83 |
| Asia | GBIF | Unknown | 14.90 | 120.85 |
| Asia | CVH | Unknown | 23.91 | 120.87 |
| Asia | GBIF | Unknown | 14.13 | 121.20 |
| Asia | GBIF | Unknown | 24.69 | 121.36 |
| Asia | GBIF | Unknown | 24.08 | 121.52 |
| Asia | GBIF | Unknown | 25.01 | 121.54 |
| Asia | GBIF | Unknown | 24.76 | 121.71 |
| Asia | GBIF | Unknown | 12.77 | 124.06 |
| Asia | GBIF | Unknown | 1.53 | 124.92 |
| Asia | GBIF | Unknown | 5.89 | 125.03 |
| Asia | GBIF | Unknown | 33.30 | 126.20 |
| Asia | GBIF | Unknown | 36.60 | 126.30 |
| Asia | GBIF | Unknown | 34.60 | 126.50 |
| Asia | GBIF | Unknown | 34.80 | 126.50 |
| Asia | GBIF | Unknown | 34.90 | 126.60 |
| Asia | GBIF | Unknown | 36.20 | 126.60 |
| Asia | GBIF | Unknown | 35.10 | 126.70 |
| Asia | GBIF | Unknown | 37.60 | 126.70 |
| Asia | GBIF | Unknown | 35.20 | 126.80 |
| Asia | GBIF | Unknown | 35.80 | 126.80 |
| Asia | GBIF | Unknown | 37.10 | 126.80 |
| Asia | GBIF | Unknown | 34.70 | 126.90 |
| Asia | GBIF | Unknown | 35.00 | 127.10 |
| Asia | GBIF | Unknown | 35.90 | 127.10 |
| Asia | GBIF | Unknown | 35.10 | 127.70 |
| Asia | GBIF | Unknown | 36.20 | 127.70 |
| Asia | GBIF | Unknown | 36.40 | 127.70 |
| Asia | GBIF | Unknown | 38.10 | 127.70 |
| Asia | GBIF | Unknown | 35.00 | 127.80 |
| Asia | GBIF | Unknown | 37.30 | 127.80 |
| Asia | GBIF | Unknown | 35.20 | 127.90 |
| Asia | GBIF | Unknown | 37.10 | 127.90 |
| Asia | GBIF | Unknown | 37.20 | 128.20 |
| Asia | GBIF | Unknown | 36.60 | 128.30 |
| Asia | GBIF | Unknown | 35.70 | 128.50 |
| Asia | GBIF | Unknown | 36.00 | 128.50 |
| Asia | GBIF | Unknown | 35.70 | 128.70 |
| Asia | GBIF | Unknown | 35.60 | 128.80 |
| Asia | GBIF | Unknown | 35.10 | 128.90 |
| Asia | GBIF | Unknown | 36.00 | 128.90 |
| Asia | GBIF | Unknown | 35.90 | 129.00 |
| Asia | GBIF | Unknown | 33.08 | 129.86 |
| Asia | GBIF | Unknown | 31.90 | 130.46 |
| Asia | GBIF | Unknown | 33.81 | 130.56 |
| Asia | GBIF | Unknown | 32.68 | 130.66 |
| Asia | GBIF | Unknown | 32.80 | 130.71 |
| Asia | GBIF | Unknown | 31.99 | 131.33 |
| Asia | GBIF | Unknown | 33.55 | 131.34 |
| Asia | GBIF | Unknown | 34.10 | 131.40 |
| Asia | GBIF | Unknown | 33.37 | 132.51 |
| Asia | GBIF | Unknown | 35.37 | 132.74 |
| Asia | GBIF | Unknown | 35.41 | 133.23 |
| Asia | GBIF | Unknown | 33.51 | 133.37 |
| Asia | GBIF | Unknown | 34.62 | 133.58 |
| Asia | GBIF | Unknown | 34.55 | 133.62 |
| Asia | GBIF | Unknown | 34.85 | 133.73 |
| Asia | GBIF | Unknown | 34.49 | 133.78 |
| Asia | GBIF | Unknown | 35.04 | 133.92 |
| Asia | GBIF | Unknown | 34.72 | 134.10 |
| Asia | GBIF | Unknown | 34.27 | 134.14 |
| Asia | GBIF | Unknown | 35.48 | 134.21 |
| Asia | GBIF | Unknown | 33.96 | 134.65 |
| Asia | GBIF | Unknown | 34.48 | 134.93 |
| Asia | GBIF | Unknown | 34.66 | 134.97 |
| Asia | GBIF | Unknown | 34.82 | 135.04 |
| Asia | GBIF | Unknown | 35.64 | 135.05 |
| Asia | GBIF | Unknown | 34.22 | 135.22 |
| Asia | GBIF | Unknown | 34.92 | 135.35 |
| Asia | GBIF | Unknown | 35.10 | 135.47 |
| Asia | GBIF | Unknown | 34.78 | 135.47 |
| Asia | GBIF | Unknown | 34.91 | 135.48 |
| Asia | GBIF | Unknown | 34.29 | 135.56 |
| Asia | GBIF | Unknown | 34.90 | 135.68 |
| Asia | GBIF | Unknown | 34.68 | 135.82 |
| Asia | GBIF | Unknown | 34.61 | 135.96 |
| Asia | GBIF | Unknown | 33.72 | 135.99 |
| Asia | GBIF | Unknown | 35.19 | 136.15 |
| Asia | GBIF | Unknown | 36.18 | 136.22 |
| Asia | GBIF | Unknown | 36.39 | 136.46 |
| Asia | GBIF | Unknown | 34.48 | 136.70 |
| Asia | GBIF | Unknown | 37.02 | 136.78 |
| Asia | GBIF | Unknown | -4.52 | 136.88 |
| Asia | GBIF | Unknown | 36.56 | 136.91 |
| Asia | GBIF | Unknown | 35.17 | 137.04 |
| Asia | GBIF | Unknown | 35.78 | 137.70 |
| Asia | GBIF | Unknown | 36.85 | 138.41 |
| Asia | GBIF | Unknown | 37.69 | 138.85 |
| Asia | GBIF | Unknown | 36.39 | 139.06 |
| Asia | GBIF | Unknown | 35.46 | 139.59 |
| Asia | GBIF | Unknown | 37.68 | 140.07 |
| Asia | GBIF | Unknown | 37.72 | 140.07 |
| Asia | GBIF | Unknown | 37.52 | 140.12 |
| Asia | GBIF | Unknown | 35.59 | 140.13 |
| Asia | GBIF | Unknown | 38.00 | 140.17 |
| Asia | GBIF | Unknown | 38.38 | 140.20 |
| Asia | GBIF | Unknown | 35.78 | 140.21 |
| Asia | GBIF | Unknown | 37.11 | 140.22 |
| Asia | GBIF | Unknown | 36.20 | 140.29 |
| Asia | GBIF | Unknown | 36.01 | 140.33 |
| Asia | GBIF | Unknown | 39.70 | 141.14 |
| Asia | GBIF | Unknown | 41.28 | 141.19 |
| Asia | GBIF | Unknown | 40.79 | 141.22 |
| Asia | GBIF | Unknown | -7.32 | 141.27 |
| Asia | GBIF | Unknown | 38.92 | 141.33 |
| Asia | GBIF | Unknown | 38.44 | 141.34 |
| Asia | GBIF | Unknown | 43.00 | 142.01 |
| Asia | GBIF | Unknown | -8.83 | 143.17 |
| Asia | GBIF | Unknown | 42.81 | 143.66 |
| Asia | GBIF | Unknown | 43.03 | 144.45 |
| Asia | GBIF | Unknown | 13.37 | 144.71 |
| Asia | GBIF | Unknown | -7.08 | 146.67 |
| Asia | GBIF | Unknown | -9.48 | 147.18 |
| Asia | Madeira et al. 2007 | B | 33.60 | 73.03 |
| Asia | Madeira et al. 2007;  Benoit 2011 | B | 26.57 | 73.84 |
| Asia | Madeira et al. 2007;  Benoit 2011 | B | 34.50 | 76.00 |
| Asia | Madeira et al. 2007;  Benoit 2011 | B | 28.62 | 77.22 |
| Asia | Madeira et al. 2007;  Benoit 2011 | B | 12.97 | 77.57 |
| Asia | Madeira et al. 2007 | B | 27.70 | 85.32 |
| Asia | Madeira et al. 2007 | A | 13.90 | 100.21 |
| Asia | Madeira et al. 2007;  Benoit 2011 | A | 5.37 | 100.24 |
| Asia | Madeira et al. 2007 | B | 19.85 | 105.67 |
| Asia | Madeira et al. 2007 | A | 10.34 | 106.36 |
| Asia | Madeira et al. 2007;  Benoit 2011 | A | -7.18 | 107.57 |
| Asia | Madeira et al. 2007 | A | 16.47 | 107.60 |
| Asia | Madeira et al. 2007;  Benoit 2011 | A | -7.29 | 110.44 |
| Asia | Madeira et al. 2007 | A | 24.15 | 120.67 |
| Asia | Madeira et al. 2007;  Benoit 2011 | BC | 37.55 | 126.97 |
| Asia | Madeira et al. 2007;  Benoit 2011 | C | 34.70 | 135.20 |
| Europe | Benoit 2011 | C | 53.37 | -9.20 |
| Europe | Benoit 2011 | C | 53.35 | -9.06 |
| Europe | Benoit 2011 | C | 55.74 | 26.79 |
| Europe | Benoit 2011 | C | 56.13 | 27.01 |
| Europe | Cook and Lüönd 1982 | Unknown | 54.00 | 22.00 |
| Europe | GBIF | Unknown | 53.61 | -10.00 |
| Europe | GBIF | Unknown | 54.96 | -4.11 |
| Europe | GBIF | Unknown | 54.35 | -3.00 |
| Europe | GBIF | Unknown | 54.39 | -2.76 |
| Europe | GBIF | Unknown | 52.07 | 0.70 |
| Europe | GBIF | Unknown | 52.15 | 1.00 |
| Europe | GBIF | Unknown | 52.05 | 1.43 |
| Europe | GBIF | Unknown | 52.14 | 1.43 |
| Europe | GBIF | Unknown | 52.50 | 1.61 |
| Europe | GBIF | Unknown | 52.59 | 1.62 |
| Europe | GBIF | Unknown | 52.49 | 1.76 |
| Europe | GBIF | Unknown | 50.76 | 6.08 |
| Europe | GBIF | Unknown | 44.91 | 8.03 |
| Europe | GBIF | Unknown | 53.42 | 14.58 |
| Europe | Madeira et al. 2007 | C | 52.22 | 21.03 |
| North America | Benoit 2011 | B | 43.75 | -112.48 |
| North America | Benoit 2011 | B | 33.76 | -94.02 |
| North America | Benoit 2011 | B | 30.71 | -84.86 |
| North America | Benoit 2011 | B | 33.79 | -82.48 |
| North America | Benoit 2011 | B | 28.86 | -82.04 |
| North America | Benoit 2011 | B | 28.06 | -81.69 |
| North America | Benoit 2011 | B | 28.17 | -81.39 |
| North America | Benoit 2011 | B | 28.42 | -81.30 |
| North America | Benoit 2011 | B | 27.23 | -80.83 |
| North America | Benoit 2011 | B | 26.93 | -80.80 |
| North America | Benoit 2011 | B | 33.45 | -80.16 |
| North America | Benoit 2011 | B | 33.30 | -80.05 |
| North America | Benoit 2011 | B | 33.20 | -79.95 |
| North America | Benoit 2011 | B | 36.68 | -78.37 |
| North America | Benoit 2011 | B | 36.42 | -77.91 |
| North America | Benoit 2011 | B | 36.51 | -77.88 |
| North America | Benoit 2011 | B | 38.12 | -77.82 |
| North America | Benoit 2011 | B | 36.42 | -77.40 |
| North America | Benoit 2011 | B | 38.83 | -77.28 |
| North America | Benoit 2011 | B | 38.91 | -76.95 |
| North America | Benoit 2011 | B | 38.82 | -76.75 |
| North America | Benoit 2011 | B | 38.68 | -75.34 |
| North America | Benoit 2011 | B | 40.47 | -75.22 |
| North America | Benoit 2011 | B | 41.13 | -73.43 |
| North America | Benoit 2011 | B | 41.81 | -72.73 |
| North America | Benoit 2011 | B | 41.47 | -72.10 |
| North America | Benoit 2011 | B | 43.41 | -70.67 |
| North America | GBIF | B | 39.01 | -122.68 |
| North America | GBIF | B | 39.82 | -122.34 |
| North America | GBIF | B | 40.47 | -122.27 |
| North America | GBIF | B | 47.37 | -122.06 |
| North America | GBIF | B | 39.15 | -121.59 |
| North America | GBIF | B | 38.19 | -120.98 |
| North America | GBIF | B | 37.22 | -119.98 |
| North America | GBIF | B | 36.33 | -119.32 |
| North America | GBIF | B | 36.13 | -118.82 |
| North America | GBIF | B | 32.79 | -117.04 |
| North America | GBIF | B | 34.91 | -117.03 |
| North America | GBIF | B | 42.48 | -116.03 |
| North America | GBIF | B | 33.00 | -116.00 |
| North America | GBIF | B | 32.70 | -115.48 |
| North America | GBIF | B | 33.00 | -115.00 |
| North America | GBIF | B | 32.83 | -114.58 |
| North America | GBIF | B | 33.50 | -112.28 |
| North America | GBIF | B | 32.23 | -111.07 |
| North America | GBIF | B | 29.45 | -101.06 |
| North America | GBIF | B | 31.39 | -100.48 |
| North America | GBIF | B | 31.50 | -99.67 |
| North America | GBIF | B | 23.03 | -99.09 |
| North America | GBIF | B | 29.95 | -99.04 |
| North America | GBIF | B | 22.77 | -99.01 |
| North America | GBIF | B | 32.83 | -98.96 |
| North America | GBIF | B | 23.29 | -98.95 |
| North America | GBIF | B | 24.57 | -98.89 |
| North America | GBIF | B | 33.44 | -98.79 |
| North America | GBIF | B | 22.99 | -98.59 |
| North America | GBIF | B | 30.56 | -98.34 |
| North America | GBIF | B | 29.29 | -98.31 |
| North America | GBIF | B | 28.48 | -98.24 |
| North America | GBIF | B | 29.88 | -98.24 |
| North America | GBIF | B | 26.04 | -98.20 |
| North America | GBIF | B | 24.88 | -98.11 |
| North America | GBIF | B | 23.96 | -98.09 |
| North America | GBIF | B | 22.91 | -98.08 |
| North America | GBIF | B | 29.65 | -98.07 |
| North America | GBIF | B | 30.38 | -98.01 |
| North America | GBIF | B | 23.03 | -97.96 |
| North America | GBIF | B | 30.39 | -97.91 |
| North America | GBIF | B | 29.86 | -97.88 |
| North America | GBIF | B | 26.06 | -97.83 |
| North America | GBIF | B | 32.29 | -97.76 |
| North America | GBIF | B | 30.25 | -97.72 |
| North America | GBIF | B | 29.50 | -97.64 |
| North America | GBIF | B | 31.02 | -97.53 |
| North America | GBIF | B | 30.16 | -97.29 |
| North America | GBIF | B | 31.60 | -97.22 |
| North America | GBIF | B | 33.78 | -97.21 |
| North America | GBIF | B | 31.90 | -97.21 |
| North America | GBIF | B | 32.72 | -97.20 |
| North America | GBIF | B | 28.72 | -97.17 |
| North America | GBIF | B | 33.07 | -97.02 |
| North America | GBIF | B | 32.64 | -96.99 |
| North America | GBIF | B | 31.56 | -96.96 |
| North America | GBIF | B | 29.91 | -96.73 |
| North America | GBIF | B | 30.31 | -96.57 |
| North America | GBIF | B | 33.74 | -96.57 |
| North America | GBIF | B | 28.95 | -96.54 |
| North America | GBIF | B | 32.90 | -96.50 |
| North America | GBIF | B | 32.18 | -96.07 |
| North America | GBIF | B | 30.63 | -96.06 |
| North America | GBIF | B | 32.53 | -95.85 |
| North America | GBIF | B | 31.76 | -95.63 |
| North America | GBIF | B | 32.81 | -95.54 |
| North America | GBIF | B | 32.06 | -95.43 |
| North America | GBIF | B | 33.04 | -95.25 |
| North America | GBIF | B | 29.85 | -95.17 |
| North America | GBIF | B | 31.39 | -95.15 |
| North America | GBIF | B | 30.73 | -95.14 |
| North America | GBIF | B | 31.93 | -94.98 |
| North America | GBIF | B | 33.04 | -94.83 |
| North America | GBIF | B | 38.86 | -94.75 |
| North America | GBIF | B | 32.88 | -94.71 |
| North America | GBIF | B | 31.45 | -94.69 |
| North America | GBIF | B | 32.38 | -94.46 |
| North America | GBIF | B | 31.71 | -94.36 |
| North America | GBIF | B | 30.78 | -94.17 |
| North America | GBIF | B | 33.31 | -94.16 |
| North America | GBIF | B | 29.94 | -94.13 |
| North America | GBIF | B | 31.07 | -94.09 |
| North America | GBIF | B | 29.83 | -94.02 |
| North America | GBIF | B | 33.70 | -93.96 |
| North America | GBIF | B | 31.71 | -93.90 |
| North America | GBIF | B | 32.50 | -93.81 |
| North America | GBIF | B | 37.69 | -93.76 |
| North America | GBIF | B | 31.18 | -93.57 |
| North America | GBIF | B | 32.71 | -93.51 |
| North America | GBIF | B | 38.27 | -93.40 |
| North America | GBIF | B | 29.87 | -93.21 |
| North America | GBIF | B | 30.21 | -93.20 |
| North America | GBIF | B | 34.57 | -93.19 |
| North America | GBIF | B | 34.22 | -93.11 |
| North America | GBIF | B | 30.74 | -93.11 |
| North America | GBIF | B | 31.95 | -93.06 |
| North America | GBIF | B | 45.53 | -92.66 |
| North America | GBIF | B | 31.58 | -92.65 |
| North America | GBIF | B | 33.30 | -92.48 |
| North America | GBIF | B | 30.97 | -92.44 |
| North America | GBIF | B | 30.74 | -92.36 |
| North America | GBIF | B | 33.05 | -92.12 |
| North America | GBIF | B | 30.55 | -91.95 |
| North America | GBIF | B | 31.00 | -91.67 |
| North America | GBIF | B | 31.58 | -91.54 |
| North America | GBIF | B | 40.58 | -91.42 |
| North America | GBIF | B | 32.67 | -91.31 |
| North America | GBIF | B | 29.72 | -91.26 |
| North America | GBIF | B | 34.02 | -91.19 |
| North America | GBIF | B | 29.88 | -91.17 |
| North America | GBIF | B | 30.29 | -91.04 |
| North America | GBIF | B | 32.34 | -90.85 |
| North America | GBIF | B | 29.30 | -90.63 |
| North America | GBIF | B | 14.48 | -90.60 |
| North America | GBIF | B | 30.30 | -90.60 |
| North America | GBIF | B | 29.65 | -90.59 |
| North America | GBIF | B | 34.76 | -90.12 |
| North America | GBIF | B | 29.92 | -90.01 |
| North America | GBIF | B | 32.48 | -89.96 |
| North America | GBIF | B | 34.66 | -89.47 |
| North America | GBIF | B | 13.63 | -89.02 |
| North America | GBIF | B | 15.66 | -89.00 |
| North America | GBIF | B | 30.65 | -88.90 |
| North America | GBIF | B | 13.74 | -88.87 |
| North America | GBIF | B | 33.58 | -88.48 |
| North America | GBIF | B | 34.36 | -88.41 |
| North America | GBIF | B | 33.23 | -88.28 |
| North America | GBIF | B | 34.94 | -88.24 |
| North America | GBIF | B | 32.87 | -88.18 |
| North America | GBIF | B | 35.18 | -88.17 |
| North America | GBIF | B | 36.82 | -88.14 |
| North America | GBIF | B | 31.76 | -88.13 |
| North America | GBIF | B | 14.91 | -87.97 |
| North America | GBIF | B | 30.77 | -87.96 |
| North America | GBIF | B | 45.31 | -87.94 |
| North America | GBIF | B | 30.68 | -87.94 |
| North America | GBIF | B | 32.52 | -87.88 |
| North America | GBIF | B | 31.69 | -87.83 |
| North America | GBIF | B | 36.05 | -87.77 |
| North America | GBIF | B | 34.90 | -87.63 |
| North America | GBIF | B | 38.16 | -87.56 |
| North America | GBIF | B | 34.83 | -87.02 |
| North America | GBIF | B | 33.47 | -86.77 |
| North America | GBIF | B | 36.30 | -86.61 |
| North America | GBIF | B | 31.33 | -86.27 |
| North America | GBIF | B | 41.05 | -86.18 |
| North America | GBIF | B | 30.64 | -86.16 |
| North America | GBIF | B | 34.52 | -86.14 |
| North America | GBIF | B | 36.29 | -85.94 |
| North America | GBIF | B | 30.45 | -85.89 |
| North America | GBIF | B | 38.28 | -85.82 |
| North America | GBIF | B | 35.02 | -85.56 |
| North America | GBIF | B | 31.86 | -85.33 |
| North America | GBIF | B | 30.79 | -85.14 |
| North America | GBIF | B | 31.26 | -85.11 |
| North America | GBIF | B | 30.81 | -84.88 |
| North America | GBIF | B | 36.08 | -84.77 |
| North America | GBIF | B | 34.16 | -84.58 |
| North America | GBIF | B | 30.53 | -84.33 |
| North America | GBIF | B | 30.23 | -84.31 |
| North America | GBIF | B | 31.53 | -84.14 |
| North America | GBIF | B | 9.98 | -84.10 |
| North America | GBIF | B | 30.34 | -83.99 |
| North America | GBIF | B | 31.56 | -83.84 |
| North America | GBIF | B | 33.04 | -83.75 |
| North America | GBIF | B | 10.92 | -83.70 |
| North America | GBIF | B | 9.33 | -83.70 |
| North America | GBIF | B | 38.68 | -83.45 |
| North America | GBIF | B | 30.61 | -83.42 |
| North America | GBIF | B | 10.33 | -83.39 |
| North America | GBIF | B | 8.70 | -83.20 |
| North America | GBIF | B | 30.10 | -83.17 |
| North America | GBIF | B | 37.22 | -82.98 |
| North America | GBIF | B | 34.76 | -82.97 |
| North America | GBIF | B | 8.83 | -82.96 |
| North America | GBIF | B | 27.98 | -82.74 |
| North America | GBIF | B | 29.86 | -82.73 |
| North America | GBIF | B | 27.84 | -82.67 |
| North America | GBIF | B | 30.16 | -82.63 |
| North America | GBIF | B | 35.54 | -82.55 |
| North America | GBIF | B | 38.43 | -82.45 |
| North America | GBIF | B | 29.10 | -82.44 |
| North America | GBIF | B | 33.83 | -82.37 |
| North America | GBIF | B | 29.54 | -82.29 |
| North America | GBIF | B | 27.13 | -82.24 |
| North America | GBIF | B | 28.52 | -82.21 |
| North America | GBIF | B | 29.92 | -82.16 |
| North America | GBIF | B | 38.84 | -82.15 |
| North America | GBIF | B | 39.03 | -82.03 |
| North America | GBIF | B | 33.45 | -81.96 |
| North America | GBIF | B | 35.75 | -81.88 |
| North America | GBIF | B | 26.54 | -81.85 |
| North America | GBIF | B | 29.52 | -81.84 |
| North America | GBIF | B | 39.08 | -81.78 |
| North America | GBIF | B | 30.31 | -81.76 |
| North America | GBIF | B | 34.60 | -81.72 |
| North America | GBIF | B | 28.94 | -81.72 |
| North America | GBIF | B | 41.39 | -81.70 |
| North America | GBIF | B | 29.97 | -81.56 |
| North America | GBIF | B | 26.09 | -81.47 |
| North America | GBIF | B | 27.94 | -81.37 |
| North America | GBIF | B | 34.07 | -81.31 |
| North America | GBIF | B | 25.99 | -81.26 |
| North America | GBIF | B | 28.72 | -81.14 |
| North America | GBIF | B | 38.12 | -81.12 |
| North America | GBIF | B | 35.26 | -81.01 |
| North America | GBIF | B | 28.58 | -80.99 |
| North America | GBIF | B | 37.37 | -80.82 |
| North America | GBIF | B | 34.38 | -80.77 |
| North America | GBIF | B | 40.19 | -80.68 |
| North America | GBIF | B | 37.05 | -80.67 |
| North America | GBIF | B | 25.42 | -80.59 |
| North America | GBIF | B | 40.48 | -80.58 |
| North America | GBIF | B | 25.94 | -80.44 |
| North America | GBIF | B | 27.13 | -80.26 |
| North America | GBIF | B | 35.85 | -80.04 |
| North America | GBIF | B | 36.78 | -80.03 |
| North America | GBIF | B | 32.99 | -79.94 |
| North America | GBIF | B | 39.63 | -79.94 |
| North America | GBIF | B | 9.12 | -79.70 |
| North America | GBIF | B | 37.05 | -79.64 |
| North America | GBIF | B | 34.86 | -79.63 |
| North America | GBIF | B | 35.29 | -79.43 |
| North America | GBIF | B | 36.41 | -79.32 |
| North America | GBIF | B | 36.00 | -78.90 |
| North America | GBIF | B | 43.03 | -78.83 |
| North America | GBIF | B | 35.76 | -78.72 |
| North America | GBIF | B | 36.31 | -78.62 |
| North America | GBIF | B | 34.32 | -78.52 |
| North America | GBIF | B | 39.22 | -78.48 |
| North America | GBIF | B | 35.74 | -77.96 |
| North America | GBIF | B | 34.26 | -77.93 |
| North America | GBIF | B | 37.42 | -77.65 |
| North America | GBIF | B | 36.38 | -77.57 |
| North America | GBIF | B | 38.29 | -77.01 |
| North America | GBIF | B | 36.38 | -76.89 |
| North America | GBIF | B | 42.45 | -76.51 |
| North America | GBIF | B | 39.47 | -76.25 |
| North America | GBIF | B | 41.91 | -76.17 |
| North America | GBIF | B | 40.83 | -73.12 |
| North America | GBIF | B | 42.06 | -70.81 |
| North America | GBIF | B | 43.70 | -70.78 |
| North America | GBIF | B | 41.67 | -70.20 |
| North America | GBIF | B | 44.16 | -69.49 |
| North America | King and Les 2016 | B | 41.77 | -72.31 |
| North America | Madeira et al. 2007 | B | 47.50 | -120.50 |
| North America | Madeira et al. 2007 | B | 31.33 | -96.32 |
| North America | Madeira et al. 2007 | B | 31.80 | -95.15 |
| North America | Madeira et al. 2007 | B | 26.13 | -80.15 |
| North America | Madeira et al. 2007 | B | 38.97 | -77.33 |
| North America | Madeira et al. 2007 | B | 39.88 | -75.19 |
| North America | Madeira et al. 2007 | B | 41.36 | -71.96 |
| North America | Madeira et al. 2007;  Benoit 2011 | B | 32.98 | -115.53 |
| North America | Madeira et al. 2007;  Benoit 2011 | B | 39.00 | -75.50 |
| Oceania | AVH | Unknown | -16.57 | 128.58 |
| Oceania | AVH | Unknown | -15.56 | 128.61 |
| Oceania | AVH | Unknown | -34.43 | 139.62 |
| Oceania | AVH | Unknown | -18.86 | 146.13 |
| Oceania | AVH | Unknown | -27.75 | 153.25 |
| Oceania | Benoit 2011 | A | -12.49 | 131.04 |
| Oceania | Benoit 2011 | A | -12.82 | 131.17 |
| Oceania | Benoit 2011 | A | -29.47 | 153.20 |
| Oceania | Benoit 2011 | A | -29.06 | 153.33 |
| Oceania | GBIF | Unknown | -17.95 | 122.20 |
| Oceania | GBIF | Unknown | -18.08 | 125.72 |
| Oceania | GBIF | Unknown | -17.20 | 126.25 |
| Oceania | GBIF | Unknown | -17.43 | 127.61 |
| Oceania | GBIF | Unknown | -15.58 | 128.28 |
| Oceania | GBIF | Unknown | -15.84 | 128.75 |
| Oceania | GBIF | Unknown | -16.08 | 128.77 |
| Oceania | GBIF | Unknown | -14.05 | 129.79 |
| Oceania | GBIF | Unknown | -13.77 | 130.45 |
| Oceania | GBIF | Unknown | -12.92 | 130.48 |
| Oceania | GBIF | Unknown | -13.67 | 130.70 |
| Oceania | GBIF | Unknown | -14.50 | 132.25 |
| Oceania | GBIF | Unknown | -13.05 | 132.68 |
| Oceania | GBIF | Unknown | -12.67 | 133.00 |
| Oceania | GBIF | Unknown | -14.95 | 133.20 |
| Oceania | GBIF | Unknown | -12.75 | 135.06 |
| Oceania | GBIF | Unknown | -19.12 | 138.57 |
| Oceania | GBIF | Unknown | -18.42 | 139.25 |
| Oceania | GBIF | Unknown | -35.13 | 139.30 |
| Oceania | GBIF | Unknown | -20.54 | 139.54 |
| Oceania | GBIF | Unknown | -34.69 | 139.59 |
| Oceania | GBIF | Unknown | -34.17 | 140.14 |
| Oceania | GBIF | Unknown | -34.28 | 140.60 |
| Oceania | GBIF | Unknown | -33.96 | 140.92 |
| Oceania | GBIF | Unknown | -34.12 | 141.93 |
| Oceania | GBIF | Unknown | -34.30 | 142.22 |
| Oceania | GBIF | Unknown | -18.25 | 142.75 |
| Oceania | GBIF | Unknown | -35.32 | 143.55 |
| Oceania | GBIF | Unknown | -19.17 | 144.43 |
| Oceania | GBIF | Unknown | -16.02 | 144.55 |
| Oceania | GBIF | Unknown | -19.75 | 144.87 |
| Oceania | GBIF | Unknown | -37.82 | 144.97 |
| Oceania | GBIF | Unknown | -18.60 | 145.00 |
| Oceania | GBIF | Unknown | -15.26 | 145.09 |
| Oceania | GBIF | Unknown | -17.92 | 145.15 |
| Oceania | GBIF | Unknown | -18.22 | 145.35 |
| Oceania | GBIF | Unknown | -19.97 | 145.58 |
| Oceania | GBIF | Unknown | -18.47 | 145.90 |
| Oceania | GBIF | Unknown | -34.20 | 146.00 |
| Oceania | GBIF | Unknown | -30.05 | 146.00 |
| Oceania | GBIF | Unknown | -18.08 | 146.00 |
| Oceania | GBIF | Unknown | -17.40 | 146.00 |
| Oceania | GBIF | Unknown | -21.02 | 146.13 |
| Oceania | GBIF | Unknown | -36.05 | 146.38 |
| Oceania | GBIF | Unknown | -38.95 | 146.45 |
| Oceania | GBIF | Unknown | -19.16 | 146.45 |
| Oceania | GBIF | Unknown | -19.83 | 147.17 |
| Oceania | GBIF | Unknown | -20.76 | 147.85 |
| Oceania | GBIF | Unknown | -20.58 | 147.92 |
| Oceania | GBIF | Unknown | -21.55 | 148.23 |
| Oceania | GBIF | Unknown | -23.46 | 148.33 |
| Oceania | GBIF | Unknown | -21.15 | 148.39 |
| Oceania | GBIF | Unknown | -20.57 | 148.48 |
| Oceania | GBIF | Unknown | -21.25 | 148.58 |
| Oceania | GBIF | Unknown | -23.62 | 149.20 |
| Oceania | GBIF | Unknown | -21.12 | 149.22 |
| Oceania | GBIF | Unknown | -24.75 | 150.08 |
| Oceania | GBIF | Unknown | -29.52 | 150.57 |
| Oceania | GBIF | Unknown | -34.13 | 150.73 |
| Oceania | GBIF | Unknown | -23.75 | 150.75 |
| Oceania | GBIF | Unknown | -33.97 | 150.87 |
| Oceania | GBIF | Unknown | -28.74 | 150.96 |
| Oceania | GBIF | Unknown | -28.60 | 151.00 |
| Oceania | GBIF | Unknown | -25.71 | 151.05 |
| Oceania | GBIF | Unknown | -33.95 | 151.10 |
| Oceania | GBIF | Unknown | -24.30 | 151.52 |
| Oceania | GBIF | Unknown | -25.58 | 151.58 |
| Oceania | GBIF | Unknown | -26.25 | 151.89 |
| Oceania | GBIF | Unknown | -32.00 | 151.92 |
| Oceania | GBIF | Unknown | -27.57 | 151.95 |
| Oceania | GBIF | Unknown | -28.87 | 152.10 |
| Oceania | GBIF | Unknown | -27.31 | 152.14 |
| Oceania | GBIF | Unknown | -25.94 | 152.33 |
| Oceania | GBIF | Unknown | -29.87 | 152.45 |
| Oceania | GBIF | Unknown | -30.82 | 152.55 |
| Oceania | GBIF | Unknown | -29.05 | 152.58 |
| Oceania | GBIF | Unknown | -28.20 | 152.64 |
| Oceania | GBIF | Unknown | -26.08 | 152.78 |
| Oceania | GBIF | Unknown | -31.41 | 152.82 |
| Oceania | GBIF | Unknown | -27.27 | 152.95 |
| Oceania | GBIF | Unknown | -28.82 | 152.97 |
| Oceania | GBIF | Unknown | -26.74 | 152.98 |
| Oceania | GBIF | Unknown | -30.26 | 153.02 |
| Oceania | GBIF | Unknown | -31.00 | 153.02 |
| Oceania | GBIF | Unknown | -26.66 | 153.06 |
| Oceania | GBIF | Unknown | -28.08 | 153.25 |
| Oceania | GBIF | Unknown | -28.64 | 153.56 |
| Oceania | GBIF | Unknown | -39.23 | 176.90 |
| Oceania | GBIF | Unknown | -19.76 | 146.84 |
| Oceania | GBIF | Unknown | -21.62 | 149.19 |
| Oceania | GBIF | Unknown | -23.89 | 151.26 |
| Oceania | Madeira et al. 2007 | B | -16.93 | 145.78 |
| Oceania | Maderia et al. 2007 | B | -38.40 | 175.72 |
| South America | Lucio et al. unpublished data | B | -27.23 | -52.03 |

Note: A, B, C, and D represent the four cpDNA genetic lineages. B is the introduced lineage.

Data Source:

1. Chinese Virtual Herbarium (CVH, http://www.cvh.org.cn/)

2. Australia’s Virtual Herbarium (AVH, <http://avh.chah.org.au/>)

3. Global Biodiversity Information Facility (GBIF, http://www.gbif.org/)

Reference:

Benoit, L. K. 2011. Cryptic speciation, genetic diversity and herbicide resistance in the invasive aquatic plant *Hydrilla verticillata* (L.f.) Royle (Hydrocharitaceae). Ph.D. Dissertation, University of Connecticut.

Cook, C. D. and R. Lüönd. 1982. A revision of the genus *Hydrilla* (Hydrocharitaceae). Aquatic Botany 13:485-504.

King, U. M. and D. H. Les. 2016. A significant new record for *Hydrilla verticillata* (Hydrocharitaceae) in central Connecticut. Rhodora 118(975): 306-309.

Madeira, P. T., J. A. Coetzee, T. D. Center, E. E. White, and P. W. Tipping. 2007. The origin of *Hydrilla verticillata* recently discovered at a South African dam. Aquatic Botany 87:176-180.

Zhu, J. 2015. Studies on molecular phylogeography and biogeography of the genus *Hydrilla*. Ph. D. Dissertation, Wuhan University.

Zhu, J., D. Yu, and X. Xu. 2015. The phylogeographic structure of *Hydrilla verticillata* (Hydrocharitaceae) in China and its implications for the biogeographic history of this worldwide-distributed submerged macrophyte. BMC Evolutionary Biology 15:95.
